# Supplementary material for: Geographic pair matching in large-scale cluster randomized trials
Source: Nat Commun. 2024 Feb 5;15:1069. doi: 10.1038/s41467-024-45152-y (PMC10844220; doi:10.1038/s41467-024-45152-y)
Supplement: Supplementary file 3 — Reporting Summary [file 41467_2024_45152_MOESM3_ESM.pdf]

nature portfolio

Corresponding author(s): Benjamin F. Arnold

Last updated by author(s): 01/3/2024

Reporting Summary

Nature Portfolio wishes to improve the reproducibility of the work that we publish. This form provides structure for consistency and transparency in reporting. For further information on Nature Portfolio policies, see our Editorial Policies and the Editorial Policy Checklist.

Statistics

For all statistical analyses, confirm that the following items are present in the figure legend, table legend, main text, or Methods section.

n/a	Confirmed	
☐	☒	The exact sample size (*n*) for each experimental group/condition, given as a discrete number and unit of measurement
☐	☒	A statement on whether measurements were taken from distinct samples or whether the same sample was measured repeatedly
☐	☒	The statistical test(s) used AND whether they are one- or two-sided<br>*Only common tests should be described solely by name; describe more complex techniques in the Methods section.*
☐	☒	A description of all covariates tested
☐	☒	A description of any assumptions or corrections, such as tests of normality and adjustment for multiple comparisons
☐	☒	A full description of the statistical parameters including central tendency (e.g. means) or other basic estimates (e.g. regression coefficient) AND variation (e.g. standard deviation) or associated estimates of uncertainty (e.g. confidence intervals)
☒	☐	For null hypothesis testing, the test statistic (e.g. *F*, *t*, *r*) with confidence intervals, effect sizes, degrees of freedom and *P* value noted<br>*Give P values as exact values whenever suitable.*
☒	☐	For Bayesian analysis, information on the choice of priors and Markov chain Monte Carlo settings
☐	☒	For hierarchical and complex designs, identification of the appropriate level for tests and full reporting of outcomes
☒	☐	Estimates of effect sizes (e.g. Cohen's *d*, Pearson's *r*), indicating how they were calculated

Our web collection on statistics for biologists contains articles on many of the points above.

Software and code

Policy information about availability of computer code

Data collection	There was no data collection software used in this secondary analysis of cluster randomized trials in Bangladesh and Kenya. Primary data were collected on handheld tablets using a custom study application programmed in Open Data Kit (ODK).
Data analysis	Analyses used R statistical software (version 4.3.2, 2023-10-31 "Eye Holes"). All code is available: https://osf.io/cxb5e/

For manuscripts utilizing custom algorithms or software that are central to the research but not yet described in published literature, software must be made available to editors and reviewers. We strongly encourage code deposition in a community repository (e.g. GitHub). See the Nature Portfolio guidelines for submitting code & software for further information.

Data

Policy information about availability of data

All manuscripts must include a data availability statement. This statement should provide the following information, where applicable:

- Accession codes, unique identifiers, or web links for publicly available datasets
- A description of any restrictions on data availability
- For clinical datasets or third party data, please ensure that the statement adheres to our policy

De-identified data and replication files required to conduct the analyses are available through the Open Science Framework (https://osf.io/cxb5e). Geographic location data required to make maps and conduct spatial analyses are not publicly available to protect participant confidentiality but are available from the corresponding author upon request, pending appropriate human subjects review and approval.

Research involving human participants, their data, or biological material

Policy information about studies with human participants or human data. See also policy information about sex, gender (identity/presentation), and sexual orientation and race, ethnicity and racism.

Reporting on sex and gender	We conducted an overall analysis across sex and gender. WASH interventions are not currently implemented based on sex or gender at the individual level; rather at the household or community level.
Reporting on race, ethnicity, or other socially relevant groupings	N/A
Population characteristics	In Bangladesh, the trial a birth cohort that was followed through approximately 2 years old, residing in rural communities within the districts of Gazipur, Kishoreganj, Mymesingh, and Tangail. The analysis included 14 different outcomes from Bangladesh with slightly different age ranges in each, which are summarized in Supplementary Information Table 1 of the paper. For example, the median (range) ages of children measured for anthropometry was 1.9 (1.4, 3.1) years; the median (range) ages of children measured for parasite infection was 2.7 (2.1, 12.0) years. The Kenya trial enrolled a birth cohort that was followed through approximately 2 years old, residing in rural communities within Bungoma, Kakamega, and Vihiga counties in Kenya's western region. The analysis included 12 different outcomes from Kenya with slightly different age ranges in each, which are summarized in Supplementary Information Table 2 of the paper. For example, the median (range) ages of children measured for anthropometry was 2.1 (1.6, 2.5) years; the median (range) ages of children measured for parasite infection was 3.4 (2.0, 15.0) years. The present study is a secondary analysis of data collected in the original trials. Arnold et al. 2013 BMJ Open https://bmjopen.bmj.com/content/3/8/e003476 includes details about community inclusion and exclusion criteria.
Recruitment	Bangladesh: Enrollment began in June 2012 for the original study trial. Each study cluster included 8 eligible pregnant women. Compounds within the same cluster were situated in proximity, allowing a single facilitator to conveniently access each participant by walking. It was possible to include multiple clusters in a village, as long as these clusters were at least a 15-minute walk apart (approximately 1 km.) from each other. Kenya (from Null et al. 2018 http://dx.doi.org/10.1016/S2214-109X(18)30005-6): Villages were eligible for selection into the study if they were rural, most of the population relied on communal water sources and had unimproved sanitation facilities, and there were no other ongoing water, sanitation, handwashing, or nutrition programmes. Participants were identified through a complete census of eligible villages. Within selected villages, women were eligible to participate if they reported that they were in their second or third trimester of pregnancy, planned to continue to live at their current residence for the next 2 years, and could speak Kiswahili, Luhya, or English well enough to respond to an interviewer administered survey. IPA staff formed clusters from one to three neighbouring villages to have six or more pregnant women per cluster after the enrolment survey.
Ethics oversight	Trial protocols were reviewed and approved by ethical review committees at the International Centre for Diarrhoeal Disease Research, Bangladesh (PR-11063), the Kenya Medical Research Institute (protocol SSC-2271), University of California, Berkeley (protocols 2011-09-3652, 2011-09-3654), and Stanford University (protocols 23310, 25863). All participants provided informed consent.

Note that full information on the approval of the study protocol must also be provided in the manuscript.

Field-specific reporting

Please select the one below that is the best fit for your research. If you are not sure, read the appropriate sections before making your selection.

☐ Life sciences ☒ Behavioural & social sciences ☐ Ecological, evolutionary & environmental sciences

For a reference copy of the document with all sections, see nature.com/documents/nr-reporting-summary-flat.pdf

Behavioural & social sciences study design

All studies must disclose on these points even when the disclosure is negative.

Study description	The present study is a secondary, quantitative analysis focused on statistical methods and reporting from two cluster randomized trials.
Research sample	The trials included birth cohorts of children followed through their first two years of life. Children were enrolled in this age range because birth to two years is the key window for diarrheal disease and growth faltering, which were the primary outcomes of the trials. Study participants were enrolled in a representative, community based sample from the study regions, where all identified women in their second or third trimester were invited to participate from the study communities.
Sampling strategy	The trials' original sample size was determined based on pair-wise comparisons between arms of two primary outcomes: length-for-age z-score (measured after 2-years of follow-up) and diarrhea prevalence (measured at 1-year and 2-years of follow-up). The trials were powered to detect a difference of +0.15 z in length-for-age and a 30% reduction in diarrhea (relative risk 0.7) for any intervention group versus the double-sized control group. Details of the sample size calculation are provided in Arnold et al. 2019 BMJ Open https://pubmed.ncbi.nlm.nih.gov/23996605/ . The very large number of clusters in each trial spread over a large geographic area (details in following paragraphs) made it an ideal study to examine the methodological aspects of geographic pair matching.

	In Bangladesh, field teams identified groups of 8 pregnant mothers in their second trimester living geographically close enough for a local health promoter to visit them regularly. This formed a cluster, and randomization was at the cluster level to enable a single health promoter to deliver a consistent intervention to all 8 pregnant mothers and their children. The field team traveled at least 1 km before starting a new cluster to prevent between-cluster spillover effects. Geographically proximate blocks of 8 clusters were pair matched and randomized to a double-sized control group (2 clusters) or one of 6 intervention groups, described below. The Bangladesh trial included 90 blocks of 8 clusters, for a total of 720 clusters .<br><br>In Kenya, the design was almost identical to Bangladesh, but clusters were slightly larger (12 pregnant mothers per cluster on average) and geographically pair matched blocks included 9 clusters rather than 8 to allow for a passive control group. In Kenya, the double-sized control group included monthly mid-upper arm circumference measurements and the passive control group included no visits to assess whether visits alone influenced outcomes (there was no difference).5 The Kenya trial included 89 blocks of up to 9 geographically pair matched clusters, but 21 blocks were incomplete so the trial enrolled a total of 702 clusters.
Data collection	Data were collected using handheld tablets programmed with Open Data Kit (ODK). Specimens were collected in study participants' homes and labeled with bar code identifiers.
Timing	Bangladesh: Between May 31, 2012, and July 7, 2013, we randomly allocated 720 clusters and enrolled 5551 pregnant women in 5551 compounds. Additional visits and specimen collection took place after approximately one year of intervention (in 2014) and after two years of intervention (in 2015).<br><br>Kenya: Between Nov 27, 2012, and May 21, 2014, 8246 pregnant women were enrolled in the study. Children were aged 2–18 months (median 12 months) at 1-year follow-up (January, 2014, to June, 2015) and aged 16–31 months (median 25 months) at 2-year follow-up (February, 2015, to July, 2016),
Data exclusions	The present analyses excluded clusters in four arms of the trial: Water (W), Sanitation (S), Handwashing (S), combined WSH. This exclusion was made to focus on the nutrition containing interventions versus control, for parsimony to create a balanced design as we were focused on methodological aspects of the design and analysis.
Non-participation	Bangladesh: 93% of children completed 2 years of follow-up in the trial. Kenya: 86% of children completed 2 years of follow-up. Supplementary Tables 1 and 2 include specific sample sizes for each outcome in each trial by control and intervention groups.
Randomization	In both trials, clusters were matched by geographic location and then randomized to control and intervention treatements. The randomization method is the focus of this paper — Methods and Results sections include many details.

Reporting for specific materials, systems and methods

We require information from authors about some types of materials, experimental systems and methods used in many studies. Here, indicate whether each material, system or method listed is relevant to your study. If you are not sure if a list item applies to your research, read the appropriate section before selecting a response.

Materials & experimental systems

n/a	Involved in the study	
☒	☐	Antibodies
☒	☐	Eukaryotic cell lines
☒	☐	Palaeontology and archaeology
☒	☐	Animals and other organisms
☒	☐	Clinical data
☒	☐	Dual use research of concern
☒	☐	Plants

Methods

n/a	Involved in the study	
☒	☐	ChIP-seq
☒	☐	Flow cytometry
☒	☐	MRI-based neuroimaging
